# Erica Ollmann Saphire: How the Study of HIV and Other Viruses Informed the Rapid Development of Vaccines and Therapeutic Antibodies Against COVID-19

**DOI:** 10.20411/pai.v7i1.515

**Published:** 2022-05-02

**Authors:** Neil S. Greenspan

**Affiliations:** 1 Case Western Reserve University, Cleveland, Ohio

## NEIL S. GREENSPAN, MD, PHD

I'm Neil Greenspan. I'm a professor of pathology at Case Western Reserve University and senior editor at *Pathogens and Immunity*. And I'm here today with Erica Ollmann Saphire, President and Chief Executive Officer of the La Jolla Institute for Immunology, to talk about her work on hemorrhagic fever viruses and SARS-CoV-2. She'll be speaking about these topics at the Keystone symposium, Lessons from the Pandemic: Responding to Emerging Zoonotic Viral Diseases. Dr. Saphire is an eminent structural biologist devoted to understanding viral pathogens and pathogenesis.

Erica, how did your work on Lassa virus and other hemorrhagic fever viruses prepare you for the switch to research on SARS-CoV-2 and the pandemic?

## ERICA OLLMANN SAPHIRE, PHD

Well, it's very much the programs that we had built before and the questions that we had been trying to answer. In order to answer the more complicated questions that one lab alone couldn't begin to address, we had galvanized international consortia to put together different labs with different expertise and different samples to answer things at a broader level.

We had had a couple years of experience of thinking through how to build larger research frameworks to enable the sample sharing and the collaboration that you know you need, within a research environment that's inherently built on competition, and how to get those systems to work. So we had already tested and refined or reiterated ways of working that allowed us to accelerate a broader, faster study that could be applied to a novel virus in a pandemic.

## NG

Having put together that kind of infrastructure, for one set of viral outbreaks was extremely valuable in preparing for the SARS-CoV-2 experience apparently.

## EOS

What was really good is the willingness of the scientists to put together their samples and their expertise and to build from the crisis something deeper and more lasting for science.

## NG

With respect to these two different virus families, are there any interesting similarities and or differences that you believe are worth making the audience aware of?

## EOS

There are a lot of similarities. One area is the immune system. We don't know what virus is going to cause the next pandemic. It might be this family or that family, it might be a re-emergence of something we already knew, it might be something entirely novel. We don't know what it's going to be, but we do know that the human immune system is what's going to have to respond. The body of knowledge that we have built as a field over the last few decades answering questions like: How do B cells respond? How do T cells respond? What are the differences between neutralizing and non-neutralizing activities in the antibodies? How do you develop a vaccine that will actually give you a longer lasting memory response? What are the arms of the immune response that correlate with protection and how are they coordinated? All of that will be applicable to the next pandemic, too. Even if we dropped down on Mars and found novel Martian viruses, the human immune system would find a way to respond.

In terms of the viruses themselves, there are a lot of similarities in how we can approach them. There are many viruses that have a membrane envelope with a fusion glycoprotein whose job is to evade your immune system, attach to your human cell and drive itself in. You're dealing with a heavily glycosylated, constantly mutating and changing, highly unstable molecule. And that has to be your target for target for antibodies and your target for vaccine design.

The body of knowledge built by studying HIV and flu and respiratory syncytial virus and Ebola and so many different pathogens — figuring out how you stabilize these kinds of molecules… how do you evaluate the kinds of glycans that are on the surface… Do the most potent antibodies block receptor binding, block cleavage, block conformational change, do something else? How do we assess which parts of these molecules don't change much through mutation? Do some of the more potent antibodies bridge epitopes? How do we develop tools, for example in structural biology, that let us appreciate the conformational complexity of these things? And what protein engineering strategies do we use to make better immunogens?

A lot of the principles about how we capture and stabilize one glycoprotein could be used for another. How HIV, RSV, and Lassa were stabilized illuminated how to stabilize SARS-CoV-2 to make an immunogen that's going to work, or a reagent for structural biology.

## NG

It's my view that with SARS-CoV-2, we're extremely fortunate because of the prior outbreaks of other coronaviruses in 2002 and 2003 and then again in 2012 with MERS, that we knew — or at least thought we knew — what the likely most important immunogenic target would be, which is spike protein. But if we had a new pathogen, where we did not know if there was more than one surface glycoprotein, and where we did not know which was the target, do you think it's fair to say it would take much longer than was the case for SARS-CoV-2 to get a vaccine? Even with mRNA technology, assuming that was going to be useful in that particular case?

## EOS

It would depend on what the pathogen is and how different it is. For SARS-CoV-2, humanity was greatly accelerated by the fact that people had begun study of coronaviruses by studying mouse hepatitis virus followed by the original SARS-1 20 years ago. And the fact that Jason McLellan had figured out how to stabilize HKU1, and that gave the first ideas to go forward for vaccines [and we built from there].

So that foundational knowledge is definitely an acceleration. We wouldn't have mRNA vaccines this year if people hadn't been tinkering with them for 20 years to figure out how to formulate them, how to package them, how to cope with innate immune responses. These things very much build on the shoulders of prior efforts.

We have a body of work now looking at adenoviral vaccines or VSV vectored vaccines or what happens if you try to chemically inactivate a virus, recombinant subunit vaccines and protein nanoparticles. For a lot of pathogens, we don't really know what the right vaccine formulation is going to be. mRNA may work for some things and not others. A VSV vectored vaccine may work for some things and not others. Having experience with approved products of many different types, and some time and relevant clinical experience around what sort of immune response you get when you use a live attenuated virus versus a protein nanoparticle versus an mRNA will let us advance multiple platforms next time.

One of the things that was pretty successful in advancing vaccines so quickly against SARS-CoV-2 was the fact that in parallel, different groups could make and evaluate different things: mRNA, protein nanoparticles, adenovirus, or other kinds of vectors and inactivated virus. If you don't know what's going to work, running many horses in the race at the same time is a good way to make sure that some are going to get over that finish line.

## NG

So, clearly, as a structural biologist, you're focused on taking biophysical information and using that to inform our efforts at development of vaccines and therapeutics. Am I correct that at the current state of knowledge, if we have a neutralizing antibody, solving the structure of the complex of that neutralizing antibody with a pathogen-derived protein, such as the SARS-CoV-2 spike protein, is typically highly informative; but, if we just had a crystal structure of such a complex without the biological experimentation, am I correct that we are not at a state of insight where we could predict even the affinity let alone the neutralization activity?

## EOS

Well, there's lots of facets there. You need the biological experimentation to tell you what antibodies you want to bother to take the time and trouble to go and image with X-ray crystallography or cryo-EM. You know something about them on the way into the structural project; you know something about the germline from which they emerge. Then the structure will show you the key residues, the key changes from that germline needed for activity. That tells you whether a vaccine might make that kind of antibody immediately or might need multiple exposures in a longer period of time.

Looking at the structure can tell you a lot of different things. It can tell you what surfaces seem to be key for the most potent activity. Do these antibodies directly block receptor binding? Is that their mechanism? Do they block fusion? Is that their mechanism? Do they bridge subunits together? How important are the glycans? Are the glycans a hindrance to limit antibody access — meaning in a next-generation vaccine, you might limit those glycans to improve how the original germline antibodies combined and start that response going — or do we need to leave those glycans in because they're key for recognition? A lot of potent antibodies might bridge some glycan to the protein surface. So the structure tells you a lot about the roadmap or what the target might be, and what modifications you might make in the antigen to better present the kinds of antibodies that look like they're most effective.

And if what you want to achieve with your vaccine is broadly protective immunity, not strain- specific immunity, you can look at the kinds of antibodies that are able to recognize multiple related viruses, or multiple strains in the same virus and see what kind of places they hit. You might want to find ways of better displaying those in the vaccine instead of the more mutagenic, more strain-specific parts.

Maybe there are parts of the antigen that you want to block out by adding a glycan. Maybe you want to take key portions that might not be as often recognized by antibodies but are successful when they are recognized. Maybe you want to just display those in some kind of protein nanoparticle. So that structure is a really good way of understanding what you're making and why, and what the end result is.

But there's a lot beyond it. So, for example, the reason we launched the large Ebola virus consortium is that we had done all the work of figuring out what is the structure of that surface antigen for Ebola and different viruses like it and where do human antibodies bind to achieve potent neutralization *in vitro*. But potent neutralization in cell culture didn't always translate to effective protection in a living thing. And so there are differences. It could be that the kind of antibodies that look fantastic in cell culture just have poor pharmacokinetics in the animal. It could be that their activity is easily recognized in the Vero cells used in your neutralization assay, but the antibody doesn't block activity and infection of a different cell type in vivo that maybe uses a different receptor or has different cleavage mechanism. So, the antibody will effectively block in that one kind of cell, but in the living thing, the viruses found a backdoor. And so you need to combine scientists with a lot of different kinds of expertise. You need structure. You need glycan analysis. You need in vivo modeling. You need to understand what you can and can't interpret from the animal models. You need people that can measure PK. You need people that can decide if these therapeutics are even manufacturable — you might have something that you love so much at an academic level, but if the protein won't express well enough or it just is not bioavailable it's not going to become an effective therapeutic.

## NG

Do you see genetic pharmacokinetic differences among antibodies of the same IgG subclass?

## EOS

So, our current Consortium for SARS-CoV-2 is evaluating antibodies and antibody-like molecules. It contains molecules in some novel formats that are multivalent to capture avidity. Some of them are sensational *in vitro*, but getting the right pharmacokinetics is challenging. Other times there are antibodies for which you might engineer them in some way, or they just happen to have some kind of hydrophobicity or some other stickiness that leads them to crossreact with a target you didn't want.

## NG

Are there any differences between what you found with how antibodies mediated immunity — let's say to Lassa virus versus SARS-CoV-2?

## EOS

There's something particular about Ebola: The viral protein that mediates entry is a transmembrane trimer, like other viruses, but it also makes this soluble, secreted, abundant dimer that can be 80 to 90% of the transcripts of the viral glycoprotein gene. So, it's abundantly dumping this dimer, and whether or not that antibody is absorbed by that dimer could be relevant for its therapeutic use. And whether or not the viral particles, used in the neutralization assay had the shape and spacing of that long spaghetti-like filovirus particle or were something smaller and rounder affected results of the neutralization assay.

The other thing that was key for Ebola virus is that it goes into the endosome first in its entry pathway. In the endosome, it's processed, and that processing removes about half the mass of the glycoprotein. There's a cleavage event that removes these heavily glycosylated domains and exposes the receptor binding core. Antibodies can be directed against epitopes in these heavily glycosylated domains. These antibodies don't neutralize in culture, meaning they don't mechanically block entry, because the virus can enter into the endosome just by macropinocytosis, whether or not the antibody is bound. After that cleavage event inside the endosome, the antibody epitope is removed, so the antibody doesn't neutralize. But those antibodies could do something else. On the surface of the virus or surface of the infected cells, the epitopes are present, and those antibodies bind just fine. By binding, they can recruit Fc effector functions and immune clearance of infected cells and pathogen. That turned out to be an important facet of protection. These antibodies were never going to neutralize, but they can recruit Fc effector functions and be protective. Other glycoproteins don't drop half their mass in the same way. But this study did point to the importance of different complementary roles of antibody and mediating protection in a whole living thing.

## NG

Can you briefly enlighten our audience as to what experiences drew you to a research career — and immunology and structural biology in particular?

## EOS

I just always thought that science was fascinating. I didn't think I'd wind up becoming an academic. I remember being five years into my X-ray crystallography PhD and still not having crystals that diffracted and I said, “I don't know what I'm going to do for the rest of my life, but it's going to be anything at all other than *this*.” But then we found crystals that did diffract, and then the information came in, and then we wrote all these papers, and then it was like, “Oh, now I get it. Now I get it.” And that's my dissertation right there, in purple.

I fell in love with structural biology because it was the most intellectually satisfying thing I'd ever seen in my life. And I still remember the day and the seat in the lecture hall I was sitting in as an undergrad at Rice when the professor explained how you could solve a crystal structure and that you would then be able to understand the XYZ coordinates in space and the B factor of how much it moved from that position of every atom in that protein. And you could see how that information was written, knit into a three-dimensional structure, and you knew how much that structure would move and breathe and change. Anything you would need to know about that molecule, you could figure out if you're just smart enough to figure out how to read it from that structure. What was fascinating from there was how to use that structural information to interpret the immune response to pathogens or the immune response to other things like cancer.

That was what originally brought me into immunology, that incredible complexity of the human immune repertoire and response and its ability to model and change and create whatever it needed to respond to whatever threat might emerge — how you might extract from that wealth of possibility something tangible, molecular meaning about how protection occurred, and how you can inspire that in a practical way with a better drug or a better vaccine. That was really the hook.

## NG

Do you have one or more scientific or career insights that you believe have been especially important in promoting your research career?

## EOS

Follow what you believe and what is most interesting to you. People ask, “What should I have my lab work on?” I say, “Something you're burning to do.” Science is hard enough; [there are] a lot of long hours. You go into it because there's something that you really want to study and you really want to learn. That enthusiasm is what's going to get you to try the 1,000th experiment when 999 didn't work. That enthusiasm is what's going to come out in your words and your grant applications and your talks, and enthusiasm is what's going to attract students and postdocs to your lab. So do what you most want to do. And just because it's not what other people find to be important and interesting right now, as long as you can find a way of conveying what you see, you might have come up with something new. We need new. If all scientists do exactly the same thing, we don't get anywhere. You need someone to find a way to ask a new question or make a new tool or look at a new system.

## NG

I'd like to thank you, Dr. Ollmann Saphire, for a highly informative and truly fascinating discussion about how structural biology can, along with other disciplines, inform our understanding of viral pathogens and how they cause infection. Thank you.

I also would like to remind everyone Keystone Symposium's Antibodies as Drugs meeting will be rescheduled, and Dr. Ollmann Saphire will be giving the keynote address at that meeting. I anticipate that it will be, as our discussion was today, extremely insightful and informative.

## SUPPLEMENTARY DATA

Supplementary materials are available at the *Pathogens and Immunity* website. Supplementary data may be provided by the authors to benefit the reader. Supplementary data are not copyedited and are the sole responsibility of the authors. Questions or comments related to supplementary materials should be addressed to the corresponding author.

